# Recent Widespread Tree Growth Decline Despite Increasing Atmospheric CO_2_

**DOI:** 10.1371/journal.pone.0011543

**Published:** 2010-07-21

**Authors:** Lucas C. R. Silva, Madhur Anand, Mark D. Leithead

**Affiliations:** Global Ecological Change Laboratory, School of Environmental Sciences, University of Guelph, Guelph, Ontario, Canada; Dalhousie University, Canada

## Abstract

**Background:**

The synergetic effects of recent rising atmospheric CO_2_ and temperature are expected to favor tree growth in boreal and temperate forests. However, recent dendrochronological studies have shown site-specific unprecedented growth enhancements or declines. The question of whether either of these trends is caused by changes in the atmosphere remains unanswered because dendrochronology alone has not been able to clarify the physiological basis of such trends.

**Methodology/Principal Findings:**

Here we combined standard dendrochronological methods with carbon isotopic analysis to investigate whether atmospheric changes enhanced water use efficiency (WUE) and growth of two deciduous and two coniferous tree species along a 9° latitudinal gradient across temperate and boreal forests in Ontario, Canada. Our results show that although trees have had around 53% increases in WUE over the past century, growth decline (measured as a decrease in basal area increment – BAI) has been the prevalent response in recent decades irrespective of species identity and latitude. Since the 1950s, tree BAI was predominantly negatively correlated with warmer climates and/or positively correlated with precipitation, suggesting warming induced water stress. However, where growth declines were not explained by climate, WUE and BAI were linearly and positively correlated, showing that declines are not always attributable to warming induced stress and additional stressors may exist.

**Conclusions:**

Our results show an unexpected widespread tree growth decline in temperate and boreal forests due to warming induced stress but are also suggestive of additional stressors. Rising atmospheric CO_2_ levels during the past century resulted in consistent increases in water use efficiency, but this did not prevent growth decline. These findings challenge current predictions of increasing terrestrial carbon stocks under climate change scenarios.

## Introduction

According to the principles of plant physiology, higher CO_2_ concentrations generally increase the ratio between carboxilation and water transpired - water use efficiency (WUE) - enhancing productivity [1], [2]. In temperate and boreal forests the synergetic effects of recent changes in climate and rising atmospheric CO_2_ are expected to further stimulate primary production [3]–[6]. Recent dendrochronological studies have identified unprecedented tree growth [7]; however, it is not clear whether this can be attributed to CO_2_ fertilization or recent changes in climate. Results from ecosystem level experiments have generally supported the CO_2_ fertilization hypothesis [8], [9], but they are limited in their ability to assess long-term mechanistic relationships between plants and the atmosphere. This, along with the fact that warming induced stress can cause tree growth decline [10], [11], leave the question of how vegetation will respond to current atmospheric changes open for debate.

Combined with isotopic analyses, dendrochronology can be used to address the limitations of the above-mentioned approaches. For example, ratios of carbon isotopes in tree rings (δ^13^C) allow us to determine atmospheric effects on stomatal conductance and plant gas exchange through time, while tree growth, measured by tree-ring width converted into basal area increment (BAI), is a reliable proxy for total carbon uptake [1], [12]. Together these two lines of evidence offer a physiologically based tool useful to decipher the past and to predict future trends of vegetation/atmosphere interactions.

Increases in WUE may be caused by either greater photosynthetic rates (carbon uptake) or by reductions in stomatal conductance and, consequently, lower transpiration [1]. In either case, changes in WUE yield changes in δ^13^C of the bulk biomass [12]. Therefore, if there is an ongoing CO_2_ fertilization effect, we should be able to detect continuous increments in WUE through changes in δ^13^C across tree-ring chronologies. Moreover, along with such increases in WUE an increasingly greater BAI should also be observed. Conversely, if BAI declines while WUE increases, it indicates that any photosynthetic advantage conferred by higher CO_2_ concentrations has not been enough to overcome warming-induced stress [13], [14]. In these cases, changes in climate should explain growth decline.

Here we determine whether systematic changes in tree growth and WUE have been occurring in temperate and boreal forests in Ontario, Canada. We sampled mature individuals of two deciduous (red oak - *Quercus rubra* L. and red maple - *Acer rubrum* L.) and two coniferous (black spruce - *Picea mariana* Mill. B. S. P. and red pine - *Pinus resinosa* Ait) species. We determined ring width and annual BAI through standard dendrochronological methods. In mature trees, ring width declines with age; thus, declining growth may be impossible to detect based on changes in ring width alone. The conversion of ring width to BAI overcomes this problem. Unlike width, age-related trends in unstandardized BAI are generally positive and this can be maintained for many decades after trees reach maturity [15]. We estimated changes in WUE by measuring carbon isotope abundances in tree rings [12]. We used multiple regression models to identify significant correlations between BAI, WUE, and climatic variables over the past century.

## Results

**Figure 1:**
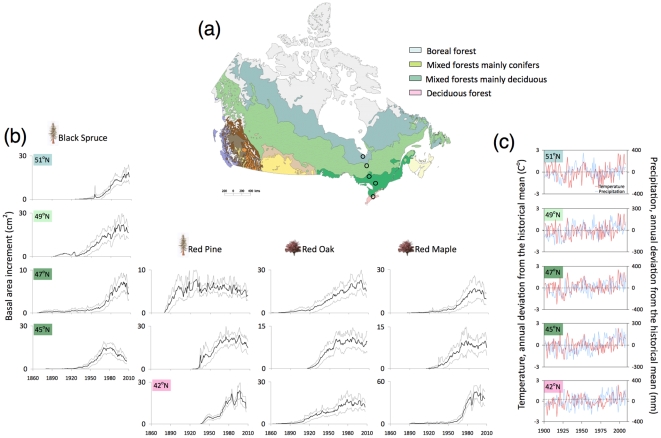
(**a**) Vegetation map of Canada and sampling sites (black circles); (**b**) average annual basal area increment (BAI) for black spruce - *Picea mariana* (Mill.) B. S. P.; red pine - *Pinus resinosa* Ait.; red oak - *Quercus rubra* L. and red maple - *Acer rubrum* L.; (**c**) past century annual temperature and precipitation deviations from the historical average mean (since 1900) for each sampling site. Species were sampled according to their range. Gray lines represent one standard deviation.

**Figure 2:**
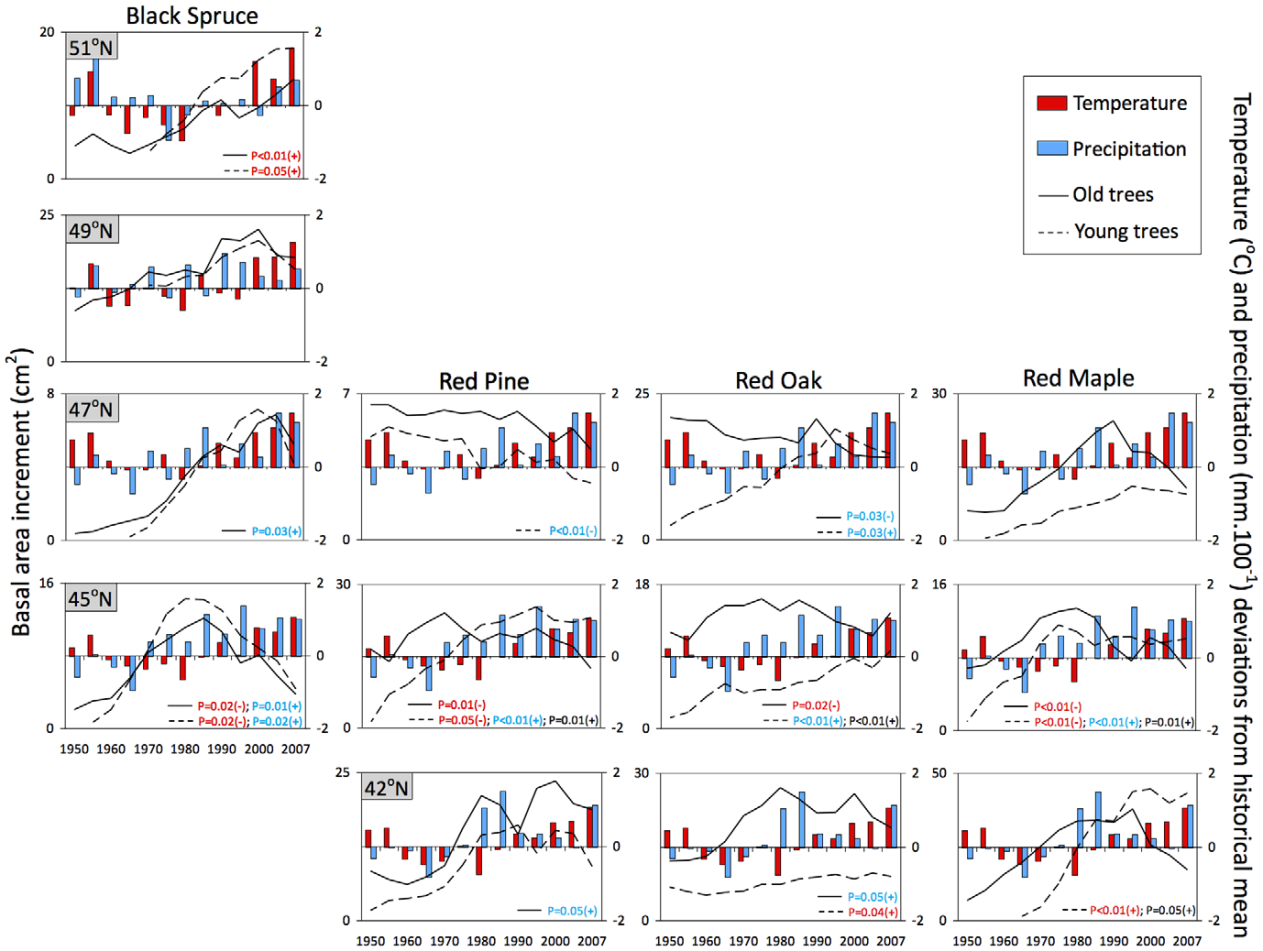
Five years averaged annual mean precipitation and temperature since 1950, deviations from the historical mean (1900–2007) and basal area increment (BAI) for the five youngest (dashed lines) and five oldest (solid lines) trees for black spruce - *Picea mariana* (Mill.) B. S. P.; red pine - *Pinus resinosa* Ait.; red oak - *Quercus rubra* L. and red maple - *Acer rubrum* L., along a latitudinal gradient in Ontario, Canada. P values represent significant correlations between BAI and temperature (red), precipitation (blue) or their interaction (black).

Trees typically showed a period of early growth suppression before a release phase, which was followed by growth decline (Fig. 1b). Overall, growth patterns for the relatively young trees resembled those of the oldest trees (Fig. 2) and cannot be solely attributed to aging. Exceptions were young deciduous trees in the lowest studied latitudes, where growth decline was not evident. We found significant correlations between BAI and recent changes in climate (temperature and/or precipitation) in most cases (Fig. 2). We observed that growth of black spruce in the northernmost site and red oak and red maple in the southernmost site was positively correlated with temperature, but we found negative or non-significant growth responses to recent warmer conditions in all other cases (Fig. 2). We only observed continuously positive trends in growth for black spruce in the northernmost studied site, with declines occurring progressively sooner toward lower latitudes (Fig. 1b). We did not observe consistent latitudinal patterns for the other species. When significant correlations were found with precipitation, BAI was generally greater during wetter periods. Red pine and red oak in the northernmost sites were exceptions with BAI negatively correlated with precipitation. The combined effect of temperature and precipitation explained growth patterns only for younger trees growing at the southern end of the latitudinal gradient.

**Figure 3:**
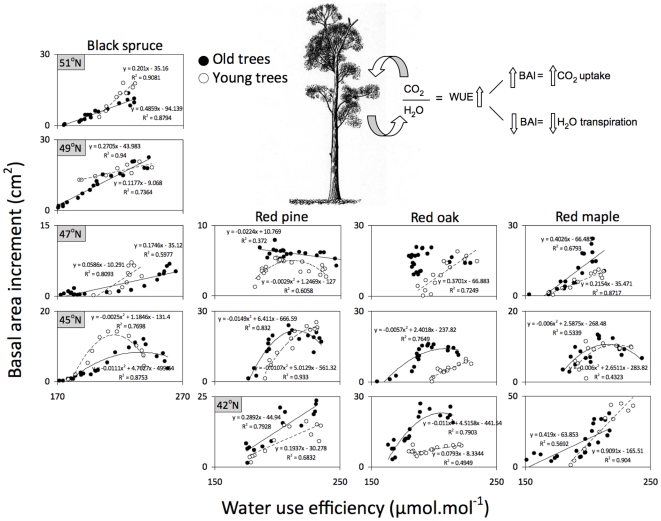
Correlations between water use efficiency (WUE) and basal area increment (BAI) for the five youngest (white circles) and five oldest (black circles) trees during the past century. WUE values were obtained from 5-year pooled rings and BAI values represent 5-year increment averages. Solid lines show significant correlation for old trees and dashed lines significant correlations for young trees (P<0.05). Positive linear relationships correspond to greater carbon uptake, while polynomial functions correspond to water stress.

**Figure 4:**
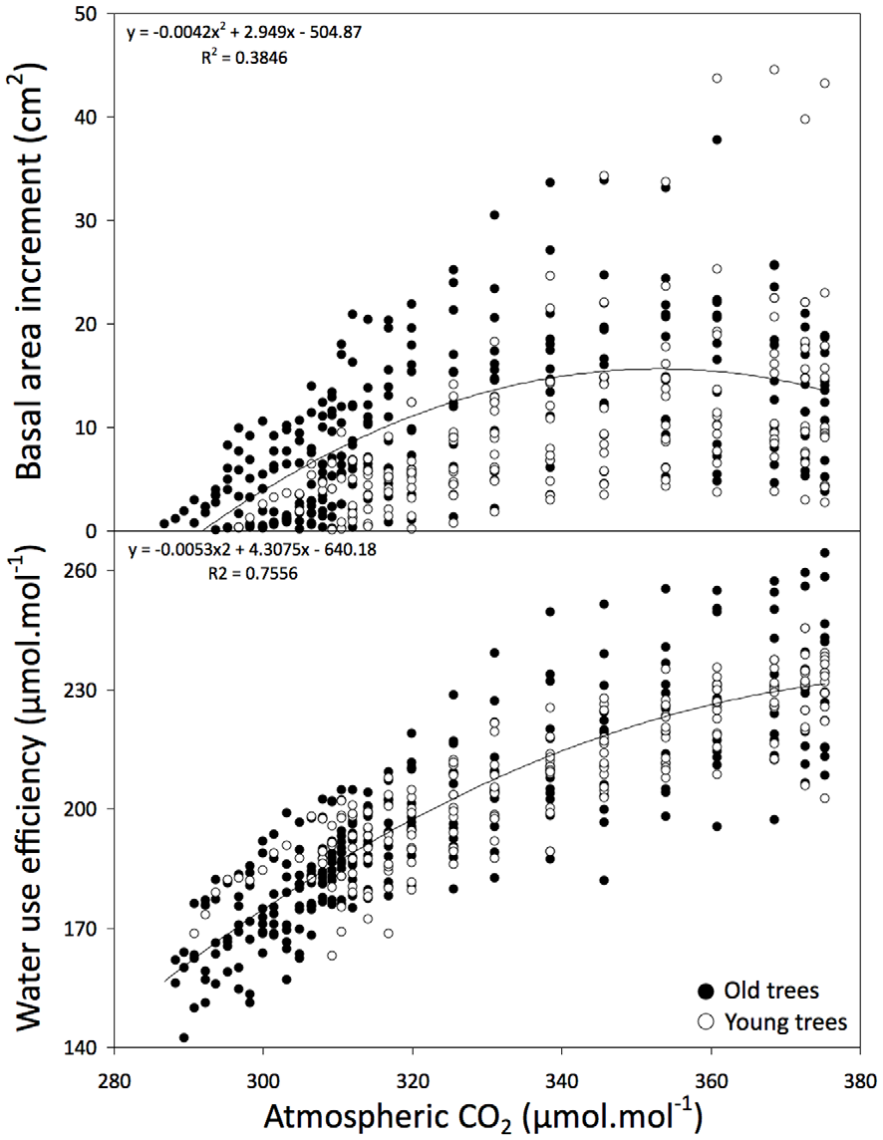
Correlations between atmospheric CO_2_ and average basal area increment (BAI) and water use efficiency (WUE) for the five youngest (white circles) and five oldest (black circles) trees from all species and sites. Atmospheric concentrations of CO_2_ summarize actual measurements and estimated values from ice cores in Antarctica from 1850 to the present [12].

Carbon isotope abundances in the tree rings confirmed that in the vast majority of cases, changes in BAI were significantly correlated with changes in WUE (Fig. 3). We found two types of relationships between WUE and BAI: linear positive and negative polynomial. When BAI responses to temperature were positive or not significant, we found linear correlations between BAI and WUE (Figs. 2 and 3). Conversely, where relationships with temperature were negative and/or precipitation had a positive influence over tree growth, WUE correlations with BAI were non-linear. Due to these different relationships, increasing atmospheric CO_2_ levels during the past century did not produce a consistent increase in BAI despite its positive influence on WUE (Fig. 4). Correlations between WUE and BAI tended to be similar among young and old trees at each studied site confirming that our findings cannot be solely attributed to aging (Fig. 3). Since WUE represents the ratio between carbon assimilated and water transpired, higher WUE could be due to either greater carbon assimilation in response to increased atmospheric CO_2_ or lower transpiration in response to water stress. The former would lead to an increase in BAI but the latter would lead to a decrease in BAI. When tree growth was negatively correlated with temperature and/or positively correlated with precipitation, BAI declined in spite of long-term increases in WUE (Fig. 3), suggesting water stress [13], [14].

## Discussion

We found recent declines in BAI for all study species and latitudes in spite of consistent long-term increases in WUE. Initial tree growth is strongly affected by stand level variations in light, nutrients, water availability and disturbance regimes [15], [16]. Accordingly, because site histories are not identical, BAI developed differently in different sites until the trees reached maturity. The growth release phase observed prior to BAI decline may have been accelerated by past century changes in the atmosphere. However, stability in BAI after growth release is the expected trend for mature trees, which should not show a decrease in BAI until they begin to senesce [15]. Since BAI and WUE trends for the relatively young trees follow the same pattern as that of the oldest trees, the recent growth decline observed here cannot be attributed to aging.

Changes in isotope ratios with ontogeny linked to developmental and microclimatic effects, also known as “age related effects”, have received attention in recent studies [12]. Such effects however are usually related to initial tree growth and cannot explain BAI decline in mature trees. Age-related effects might represent part of the WUE increases that we observed here, but ontogenic processes are known to be minor when compared with long-term effects of changes in atmospheric CO_2_ and climate [12]–[14]. Co-occurring ontogenic related influences include: assimilation of δ^13^C-depleted air near the forest floor [17], changes in irradiance and photosynthetic capacity [1], and changes in the vapor pressure deficit with height in the canopy [18], none of which can explain a ∼53% WUE change observed during the past century. Similar increases in WUE were recently reported for species of tropical, temperate and boreal trees, consistent with theorized CO_2_ fertilization effects [13], [14], [19], [20], [21]; however, our observations show a recent decrease in BAI in spite of long-term increases in WUE.

It is possible that changes in BAI are not related to CO_2_ fertilization effects, but driven by warmer temperatures. Strong increases in tree growth in colder conditions above (but not below) the tree line and growth declines associated with warmer climates at lower altitudes have been recently reported [7], [13]. In our study we observed continuously positive trends in growth only for black spruce in the northernmost (coldest) studied site, with the onset of declines occurring progressively sooner (1970 at 43°N) towards lower latitudes. Deciduous species did not show the same latitudinal pattern and tended to have lower decline rates than conifers. Growth of black spruce in the northernmost site and red oak and red maple in the southernmost sites represented the only cases where we did observe significant positive growth in response to recent higher temperature (Fig. 2). Combined effects of temperature and precipitation were significantly related to growth patterns, but only for young trees growing at low latitudes (Fig 2). BAI declines were not evident for young deciduous trees at the lower end of the latitudinal gradient (Fig 2) and accordingly they represent the outliers observed in Fig 4. If this trend persists, a reconfiguration of species ranges, as previously theorized through northward migration of the temperate/boreal ecotone [9], [22], [23] may hold true.

In some sites WUE and BAI were linearly and positively related, suggesting that growth decline is not always related to water stress. These were the very same sites where growth trends were not negatively related with warmer conditions and/or positively related with precipitation. In these cases BAI decline would be necessarily linked with reductions in WUE, meaning loss of sensitivity to CO_2_. Secondary effects of changes in climate, such as changes in seasonality, snowmelt time and differential growth/climate relationships could explain this loss of sensitivity [11]. In addition, warmer climates may also cause a deceleration in tree growth by increasing rates of respiration. However, since growth is not limited by carbon and acclimation of respiration is likely occurring, respiration cannot explain growth declines [24]. It is also possible that warming-induced canopy dieback, recently observed in western US temperate forests [25], could produce such effects. However, our sampling criteria focused on healthy trees without signs of canopy damage.

There are several non-climatic explanations for tree decline, some of which we can rule out for our study. Autogenic succession and endogenous increasing light competition are unlikely explanations because we sampled canopy trees and the species studied represent diverse successional groups. Rising tropospheric ozone concentrations have been shown to damage the photosynthetic apparatus, reducing plant sensitivity to CO_2_ and promoting growth decline, but this would require extremely high concentrations of O_3_ near the canopy. Additionally, deleterious effects of O_3_ on carboxilation rates seem to be restricted to young seedlings [26] and O_3_-induced effects at the leaf level do not necessarily correspond to reduced tree growth or WUE [27].

A possible explanation for growth decline in the cases where we did not find evidence of water stress is progressive nutrient limitation. Recent ecosystem level experiments have demonstrated this process in US temperate forests [8], [28]. Photosynthetic capacity is mostly limited by nutrient limitations, which may reduce WUE and BAI [1], [24]. Although tree growth is generally consistent with species niche differentiation, soil-plant feedbacks can have a greater influence on growth than species-specific differences at the ecosystem level [29], [30] and yield convergent signals such as the ones observed here. Nitrogen has been considered to have a preeminent role in regulating tree growth and other aspects of ecosystems beyond the species level [8], [28]. The relative importance however of nitrogen compared to other nutrients may have decreased because of atmospheric deposition and other nutrients (e.g., calcium and phosphorus) appear now to be of equal importance as nitrogen in determining limits to ecosystem productivity [31] and tree growth rates [16], [32].

Most of our study sites are located within the Canadian Shield that is characterized by very thin organic soil lying on granite bedrock with many bare rock outcrops formed from glacial retreat. Trees growing on these soils should have higher sensitivity to drought stress and nutrient limitation than on deeper, well-structured soils found elsewhere. For instance, the common subsurface water runoff immediately after rainfall events within the Shield can lead to water stress. Additionally, despite high levels of inorganic nutrients in throughfall, subsurface runoff losses are nearly inexistent, showing that biological demand for these nutrients is greater than that available in soils [33]. Thus, favorable water balance and a rapid nutrient turnover from biomass to labile forms are essential to maintain steady productivity in these environments.

In conclusion, we suggest that either drought stress or nutrient limitation could explain the recent growth decline reported here. Demand for other resources may have a greater effect on tree growth than a positive influence of new atmospheric conditions such as elevated CO_2_. Long-term experiments to test soil-plant feedbacks and the effects of atmospheric changes on tree physiology should provide a more in-depth understanding of why expected increases in tree growth are not found. This, in combination with new forest models that take into account multiple stressors will improve our knowledge of forest dynamics as well as our estimates of terrestrial carbon stocks under climate change scenarios.

## Materials and Methods

### Sampling design

We chose mature stands in preserved areas with no recent history of anthropogenic disturbance. We sampled between twenty and thirty-five living trees of black spruce (*Picea mariana* Mill. B. S. P.), red pine (*Pinus resinosa* Ait), red oak (*Quercus rubra* L.) and red maple (*Acer rubrum* L.) at five different latitudes according to their range (Fig. 1a). This latitudinal gradient represents the transition between the southern deciduous (Carolinian) forest to the south and the boreal forest to the north in Ontario, Canada. We sampled trees at: Long Point Waterfowl & Wetland Research Station (42°42′N; 80°21′W); Algonquin Provincial Park (45°35′N; 78°21′W); Wolf Lake Forest Reserve and Fairbanks Provincial park (46°47′/51′N; 80°/81°37′/43′W); Esker Lakes Research Station (49°10′N; 80°5 9′W) and a black spruce dominated forest a few kilometers west of Moosonee municipality (51°17′N; 80°38′W).

### Analysis of wood cores

We obtained two to four wood cores for each tree with an increment borer (5.1 mm). We mounted the cores on supports where they were air-dried and polished with sandpaper from 60 to 600 grains. We counted and measured the growth rings and dated the cores using the software Windendro. We assigned the calendar age of the growth rings according to Schulman's criteria [34]. In order to check dating accuracy, we compared ring-width time series within trees at each site. This gives a similar pattern of growth in a given population, allowing us to identify annual rings for each tree and to correct primary errors caused by partial or false rings. Dendrochronological measurements were performed at the Climate and Ecosystem Dynamics Research (CEDaR) Laboratory of the University of Guelph.

In mature trees, ring width declines with age and therefore cannot be considered an accurate measure of tree growth [12]. For instance, if a declining growth trend is suspected, it may be impossible to detect it on the basis of changes in width alone. The conversion of ring width into BAI overcomes this problem. Unlike width, age-related trends in unstandardized BAI are generally positive. Therefore mature trees should yield linearly positive trends in BAI over the past few decades [15]. Here, the conversion of ring width into BAI is shown annually (1 year = 1 full ring, late and early wood), assuming that increment was uniform along each ring:
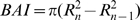
(1)where *R* is the tree radius and *n* is the year of tree ring formation.

We further investigated all individual trees for changes in WUE as shown by changes in wood carbon isotope composition through time. For isotopic analysis we used the five youngest and five oldest trees of each species at each site, resulting in a total of 130 trees. For each of these trees we pooled rings (5-year blocks) using a microscope and separated them using a thin sharp blade. Combining rings for isotopic analysis is a common practice that allows adequate sample homogenization, yields enough material for the analysis and reduces processing time. We coarsely ground wood samples in a microgrinder until the particles achieved a thickness of 1.5 mm and transferred them to Eppendorf tubes. We took an aliquot from each of the Eppendorf tubes and weighed each on a microbalance (weight range 2–3 mg). We transferred each aliquot to a tin stain capsule (3 mm diameter and 8 mm height, Elemental Micro-analysis, Milan, Italy), sealed by compressing the cup and then stored in a plate ready for analysis. We prepared one standardized aliquot at every 10^th^ aliquot and a “blank” (empty tin cup) every 40^th^ aliquot. We prepared standards in the same way as the wood samples by weighing between 1 and 2 mg of semolina. We placed all samples in an automated elemental analyzer (Euro-EA-Elemental Analyzer, Eurovector, Milan, Italy) connected to a continuous flow isotope ratio mass spectrometer (Isoprime, GV, Manchester, England) generating results with 0.1‰ of accuracy.

To investigate WUE using this type of analysis we rely on the natural existence of both carbon 12 (C^12^) and carbon 13 (C^13^) stable isotopes in the atmosphere. The CO_2_ molecules contain these isotopes in the proportion of 98.89% C^12^ and 1.11% C^13^; however in all plant tissues carbon isotope ratios are variable and the C^13^ abundance relative to C^12^ is usually expressed as δ^13^C:
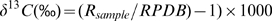
(2)in which R*_sample_* and *RPDB* represent the ^13^C/^12^C ratios of the sample and PeeDee international standard respectively [1].

We compiled historic atmospheric δ^13^C levels, and estimated values for the recent atmospheric concentration of CO_2_, from McCarroll & Loader [12] who summarized high precision records of the long-term atmospheric δ^13^C measured from an ice core in Antarctica. We determined the *C_i_* (intercellular CO_2_ concentration), eliminating changes in atmospheric isotopic composition effects on plant carbon, using Francey & Farquhar's [1] equation:
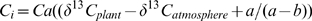
(3)where *δ* ^13^ *C_plant_* and *δ* ^13^ *C_atmosphere_* are the plant and atmospheric isotopic carbon ratios, respectively, *a* is the diffusion fractionation across the boundary layer and the stomata (≈4.4‰), *b* is the RuBisCo enzymatic biologic fractionation (≈27.0‰), *C_i_* and *C_a_* are the internal and external partial pressure of CO_2_, respectively.

Carboxilation rates (total carbon uptake) and plant water loss (transpiration) are, like carbon isotope discrimination, controlled by photosynthesis and stomatal conductance (see Fick's first law for further explanations). Since the ratio of carbon fixed to water loss is the definition of water use efficiency, we expressed the carbon ratio values in terms of changes in water use efficiency [12] by the following equation:
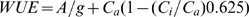
(4)where the intrinsic water use efficiency (WUE) or the rate of CO_2_ assimilation by the plant (*A*) to its stomatal conductance (*g*) is a function of *C_i_* and *C_a_*, internal and external partial pressure of CO_2_, considered that *g* for CO_2_ molecules is 0.625 times *g* for leaf conductance to water vapor. All the isotopic analyses were performed at the Laboratory of Stable Isotope Ecology (LSIETE) of the University of Miami.

### Data treatment and statistical analysis

We calculated the average annual BAI and standard deviation values for each species at each site. Growth patterns were consistent regardless of changes in tree age and/or tree size. The five youngest and five oldest trees of each species at each site showed similar radial growth (BAI) patterns through time. We used multiple regression models and analyses of variance (ANOVAs) to identify significant correlations between BAI (dependent variable) and temperature, precipitation and their interaction (independent variables) since 1950, when most trees had already reached maturity. As typically used to interpret climate data, changes in temperature and precipitation are presented as the deviation from the historical mean (1901–2007) (Figs. 1c, 2). Historical data on precipitation and temperature were provided by the Ministry of Natural Resources of Canada and can be accessed through the website: http://cfs.nrcan.gc.ca/subsite/glfc-climate/meansurfaces.

We observed that changes in BAI were linked with changes in WUE and tested the significance (P<0.05) of these relationships for the youngest and oldest trees of each species at each site during the past century using least-squares and non-linear regressions. Using non-linear regressions we determined BAI and WUE responses to increasing atmospheric CO_2_ for young and old trees for all species across sites. We performed all regressions using 5-year BAI average values to match the WUE values calculated form pooled rings (5-years blocks) as previously described. We performed all statistical analysis using JMP software for Macintosh, version 8.0.
